# The Indonesian consumer perspective on the massage industry: A conjoint analysis approach

**DOI:** 10.1371/journal.pone.0308098

**Published:** 2024-08-29

**Authors:** Yogi Tri Prasetyo, Maela Madel L. Cahigas, Abel Phillip Tan, Lilian Evangelista Kurniawan, Stevano Nyoto Prawiro, Reny Nadlifatin, Ma. Janice J. Gumasing, Irene Dyah Ayuwati

**Affiliations:** 1 International Bachelor Program in Engineering, Yuan Ze University, Chung-Li, Taiwan; 2 Department of Industrial Engineering and Management, Yuan Ze University, Chung-Li, Taiwan; 3 School of Industrial Engineering and Engineering & Management, Mapua University, Manila Philippines; 4 Department of International Business Engineering, Petra Christian University, Surabaya, Indonesia; 5 Department of Information Systems, Institut Teknologi Sepuluh Nopember, Kampus ITS Sukolilo, Surabaya, Indonesia; 6 Department of Industrial and Systems Engineering, Gokongwei College of Engineering, De La Salle University, Manila, Philippines; 7 External Research & Development, University of Surabaya, Surabaya, Indonesia; Endeavour College of Natural Health, AUSTRALIA

## Abstract

The massage industry has been in the market for more than thousands of years. Consumers purchase massage services to treat illnesses, alleviate pain, or improve well-being. Despite the popularity of this industry and the benefits it entails, consumers’ preferences vary and massage parlors’ stakeholders have inconsistent market segmentation. Therefore, the purpose of this study was to investigate consumer preferences toward massage services offered by massage parlors in Indonesia through conjoint analysis. Conjoint analysis’ orthogonal design concentrated on stimuli preferences and it was further supported by generating 1.000 consistency and reliability based on Kendall Tau’s holdout. A total of 212 respondents answered the online questionnaire voluntarily. The results showed that the Google rating was the most important attribute (68.5%), followed by the gender of the massage therapist (12.4%), the type of massage (9.6%), the type of room (4.5%), the duration (3.6%) and the price (1.4%). Moreover, this research assessed 27 stimuli and found that the best combination was employing female massage therapists, IDR 100k-150k price every session, 90 minutes massage duration, couple room with two beds, acupuncture and cupping massage type, and massage parlors with greater than 4.6 Google review rating. This present research was one of the first studies that explored unique and holistic massage services through conjoint analysis. Unlike the previous studies that only focused on one massage service attribute or did not provide specific levels for evaluated attributes, the conjoint’s orthogonal design delivered a new perception of prioritizing both consumers and the business side as some would only focus on one or the other. Also, the findings could be useful for massage parlors’ stakeholders in developing marketing strategies, enhancing operational strategies, and promoting customer satisfaction. Marketing tactics such as promotional discounts would inspire customers to provide an optimistic Google review. Business owners were encouraged to focus on giving the best experience starting with a well-trained massage therapist, clean and hygienic rooms, and quality materials. These theoretical and practical implications aid in building the massage parlor’s credibility that could be perceived positively by consumers.

## Introduction

The massage industry emerged in 460–377 (Before Common Era), more than thousands of years ago, and has been in the marketplace regardless of geographical location [1]. It stayed in the market due to its holistic impact on consumers’ health. Indonesia is one of the countries that continuously maximizes the services offered by massage parlors. Based on business perspectives, Indonesian citizens have become wealthier and more productive over the past few decades with the rise of service-based jobs in Indonesia. Most specifically, the demand for massage and personal care services increased every year and is projected to continuously increase until 2027 [2]. The massage industry is forecasted to grow 12.29% annually over the next 3 years in Indonesia, resulting in a projected market volume of $4.78 billion by 2025 [2]. To capitalize on the growth of these service industries, stakeholders of massage parlors must understand the most crucial factors that influence the popularity of their services. One of the key aspects revolving around massage parlors is the offered massage packages.

A massage package is a selection of massage that consumers can choose freely. It includes the gender of the massage therapist, the cost of massage therapy, the massage duration, the type of room, the type of massage, and feedback from past consumers. These choices are taken into account because they influence customer behavior, and eventually preferences. For instance, it is common for consumers to favor female massage therapists over male therapists due to societal norm issues [3, 4]. The duration, type of room, and type of massage are correlated with the cost. A massage type with a longer duration will cost more than a massage that requires a shorter time. Since consumers have the power to choose from available rooms, they can pick a room that best suits their relaxation preferences and financial capabilities. A private room is more expensive contrary to the common room. Moreover, prices for types of massage vary depending on the needs of consumers, especially since each massage type can benefit distinct areas. Price is often investigated in consumer behavior studies because consumers are sensitive to prices [5]. On one hand, it was uncommon for academicians to consider consumers’ direct feedback based on Google reviews. Hence, these attributes are evaluated in the present study.

These aforementioned attributes promote self-care because they aid in consumers’ relaxation by choosing a massage service attribute that fits their needs. A past study revealed that massage experimental studies can be applied to individuals, pairs, or groups [1]. This current research will focus on individual consumer preferences to establish a point of reference in the context of the massage industry in Inodnesia’s academe setting. It is deemed important to establish individual preferences first because group identity is not a good predictor as one person may influence another person’s perception. In addition, there is a need to address gender issues in the massage industry. Massage therapists in Canada experienced discrimination as most businesses prefer female massage therapists [3]. Since most massage consumers are women, the discomfort of the opposite gender in one massage room arises. However, the study acknowledges cultural differences and job professionalism. Thus, the present study may lead to a different finding. The selected subjects of the study can be experimented with as well. For instance, some studies investigated the massage perceptions of health science students [4], male athletes [6], postpartum mothers [7], people with dementia [8], post-stroke survivors [9], and females undergoing maintenance hemodialysis [10]. It can be depicted that these subjects have health concerns and have been thoroughly investigated by researchers. Hence, the present research creates a novel approach by catering to the general perspective of massage consumers with or without health issues.

Furthermore, the researchers applied conjoint analysis because it aims to predict the behavior of consumers toward the business model’s services. Conjoint analysis can determine the importance of services’ attributes and levels [5]. It is a distinct statistical technique geared to structure consumer market segmentation and massage parlor operations. Thus, it helps increase the quality of massage parlors by allocating resources and maximizing them effectively. This technique benefits both the business owners and the massage parlor’s consumers. However, none of the past studies utilized conjoint analysis in the context of the massage industry. For example, Ong et al. [5] employed conjoint analysis to determine the preferences of gym-goers. Another example was the selection of COVID-19 vaccine preferences written by Sun et al. [11]. The first two examples contributed to health allied-relate studies in the conjoint analysis technique but did not discuss massage services. Lastly, Guevarra et al. [12] centered their research on lean six sigma coaching. Although the past study opened a new industry application, it focused on selected project procedures and overlooked stakeholders’ perspectives alongside the imminent project outcomes.

The goal of this study is to analyze consumer preferences toward massage services offered by massage parlors in Indonesia by utilizing conjoint analysis incorporated with six attributes (massage therapist’s gender, price of each massage session, duration of the massage, type of room, type of massage, and Google review rating). In this study, conjoint analysis is structured by creating an orthogonal design that measures the preferences of consumers on each stimulus. Stimulus is created by generating different combinations from the aforementioned attributes encompassing varied levels of measurement. Thus, the authors would like to explore the importance of these attributes and their corresponding levels.

Despite the benefits instilled by massage services, this industry was rarely examined in Indonesia and in a credible academic setting. The existing database mostly reflects studies from Canada [3, 6], U.S. [4, 13], Malaysia [7], and Taiwan [8]. Moreover, other researchers settled for a lack of well-established statistical techniques. For instance, Baskwill and Vanston [3] only utilized descriptive statistics and they focused on interpreting massage therapy results in qualitative form. The main disadvantage of descriptive statistics is its lack of qualitative measurement that interpolates massage service attributes. Smith et al. [13] found a significant difference between pain relief and massage techniques through ANOVA. However, ANOVA can only find the significance of mean differences and not the most preferred massage attributes. While Munk et al. [4] combined descriptive statistics and ANOVA, their results were limited to one independent variable (gender). This past finding undermined the effectiveness of determining massage consumer preferences. In the present study, the authors utilized past studies as a research gap and a reference point of conjoint design. Hence, this study provided a more comprehensive combination of mathematical and theoretical approaches by creating an orthogonal design through conjoint analysis. Furthermore, the conjoint analysis was utilized to represent the most recent data on massage service consumers in Indonesia. These arguments were only present in the current research, which local and international studies overlooked. Considering the research gap revolving around Indonesia’s massage industry, this present research is one of the first studies that utilize conjoint analysis to find the best stimulus combination or massage service package applicable to Indonesian consumers.

Apart from academic contribution, this study can be useful for massage parlors’ stakeholders in developing marketing strategies, enhancing operational strategies, and promoting customer satisfaction. Once consumers’ preferences are identified by stakeholders, they can easily customize massage services to be offered to their customers. They can improve staff attitudes and hire massage therapists strategically. The interior design and clinic structure are also encouraged for further enhancement since some attributes cater to aesthetics and privacy. In addition, the findings of the study would aim to increase positive brand awareness to maintain customer loyalty and attract new customers. Furthermore, the findings and methodology can be applied to other health service-based jobs geared toward consumers’ well-being. Since health is the most crucial aspect of life, consumers put health-related services on top of their priority. Researchers with medical backgrounds can perform healthcare experimental studies that are geared toward real-life applications from an identified sample size.

## Literature review

### General perspective

The traditional consumer perspective where buyers purchase and review products in-store has evolved to digital consumption. Despite the evolution, these two worlds are still evident separately since some consumers prefer to have both setups or either of the two. One study noted a significant difference in actual rating experience compared to Google ratings [14]. This supports that personal experience upholds superiority in customer feedback for items that require human interaction. On one hand, the power of digital consumption is elevated through accessibility as most consumers purchase a product through online reviews [15]. Therefore, understanding consumer behavior is redefined as a sustainable approach because stakeholders can identify the motivations and preferences easily [16]. For instance, a past study helped stakeholders focus on enhancing the brand’s reliability by addressing negative feedback from consumers [17]. This proactive approach would build trust by gaining positive feedback, and eventually lead to an increase in profit. Apart from businesses that focus on producing products, some venture into the service industry. Specifically, the present research assessed consumer behavior in availing of massage parlor services. Massage therapies are performed by trained professionals since they may customize the package based on the consumer’s health [13].

Massage services are recommended by health practitioners to alleviate patients’ pain and improve welfare [4]. While it is common for athletes and old adults to avail of massage services, some studies noted positive therapeutical impacts of massage for pregnant and postpartum women [6, 7]. Nonetheless, normal people without the need for immediate medical intervention are also encouraged to visit a massage parlor to maintain good health and self-care [1]. These circumstances demonstrate that people of different nationalities, cultures, and ages can maximize massage services. But it should be known to consumers that the best massage type can only be maximized through proper diagnosis and assessment from healthcare practitioners. However, there is a mixed reaction among consumers because of varying massage parlor services [3, 4].

Previously, researchers focused on assessing the massage effects on consumers with medical issues. In Taiwan, Liu et al. [8] studied the effects of massage on patients with dementia. Cabanas-Valdés et al. [9] investigated the effectiveness of massage therapy in improving the condition of stroke survivors in Spain. In addition, Ghasemi et al. [10] compared the benefits of aromatherapy and foot reflexology on the severity of restless leg syndrome among hemodialysis female Iranian patients. While these past studies contributed to the medical field’s practical implications, they solely focused on experimental design. The present study focused on the perceptions of both patients and consumers, which positively influence the internal business operations of massage parlors. Due to the nature of experimental studies, they were limited to basic statistical analysis. In the current study, the researchers expounded on consumers’ perceptions through conjoint analysis, which was believed to be the most appropriate method to dig deeper into massage parlors’ services.

### Theoretical framework

**Fig 1:**
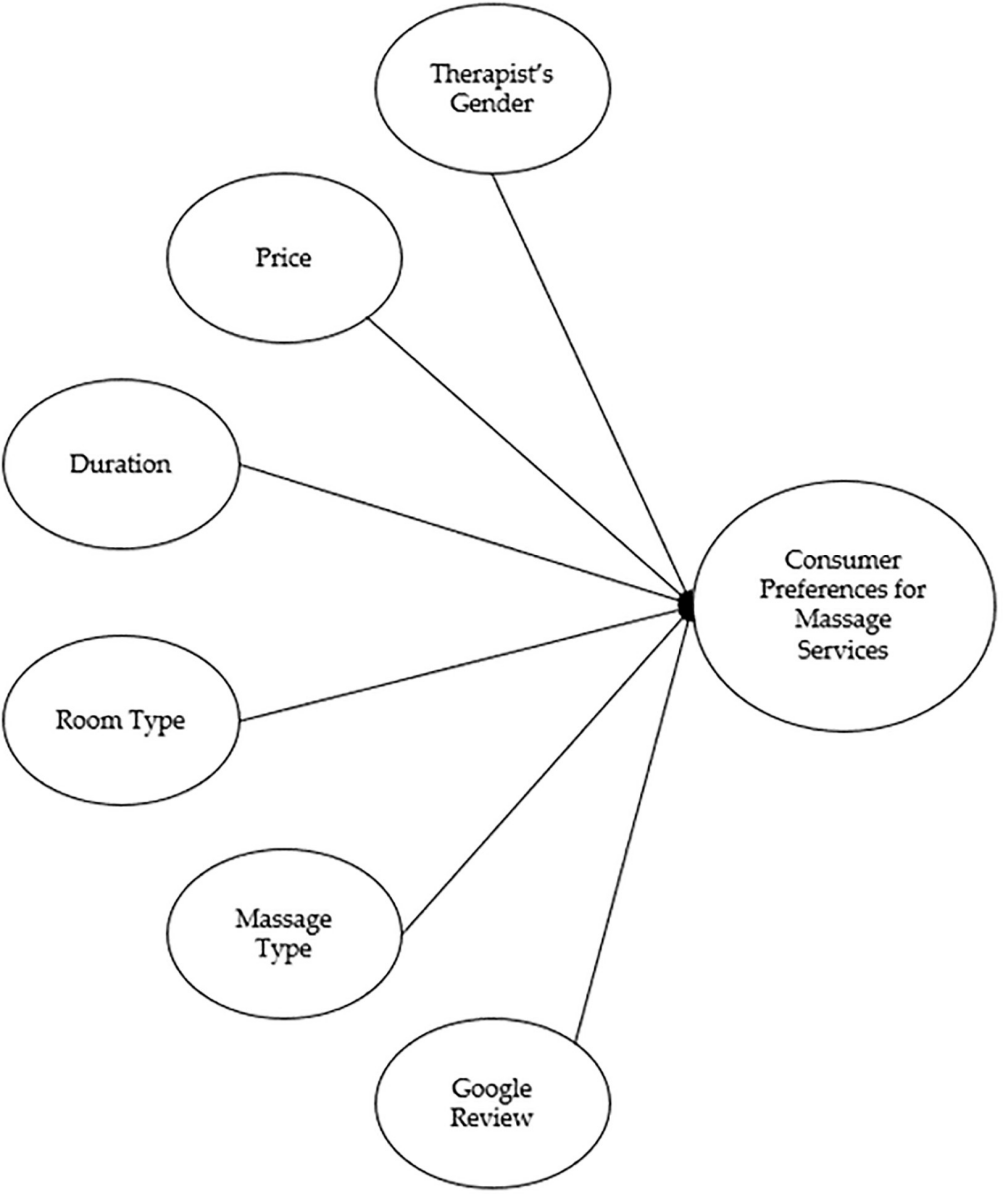
Theoretical framework of the conjoint design.

This study evaluated a total of six massage service attributes which are as follows: (1) massage therapist’s gender, (2) price of each massage session, (3) duration of the massage, (4) type of room, (5) type of massage, and (6) Google review rating. All of them are identified to have a connection with consumer preferences as displayed in Fig 1.

The massage therapist’s gender refers to the gender role associated with the subject’s exhibited behavior. It is necessary to evaluate gender because the communication process contributes to massage outcomes [13]. A study experimented on the significant differences between the opposite gender of massage therapists compared to the gender of the consumer and those consumers with similar gender to the massage therapist [13]. Similar to another study’s approach, they investigated the effects of different massage therapist genders on the subject’s mental status [18]. Both studies found insignificant effects of gender, however, it was noted that female massage therapists would elevate the mood of massage consumers [13, 18]. Meanwhile, another study mitigated gender bias by purposely selecting female massage consumers [10]. Based on their findings, male massage consumers might have different preferences for massage services compared to female massage consumers. This stems from the purpose of availing massage services as men mostly utilize massage for health purposes while women mostly avail it for relaxation.

The theoretical framework’s price pertains to the cost that a consumer pays for a massage service. Price or cost is one of the top most searched keywords that help researchers analyze consumer preferences [15]. The past study’s data scraping technique aided the current study in adding price as one of the conjoint attributes. Their insights are beneficial considering that they evaluated research published within a 10-year period. Since pricing strategy only varies between low and high, consumers tend to classify massage as a low-priced or high-priced service. Most of the clustered consumers from a past study indicated that they prioritize a good ratio between quality and price [16]. However, a few consumers from the younger generation would prioritize low-priced products because of financial constraints [16]. The presence of these conflicting findings can be closed by taking the general price perception of consumers alongside other attributes affecting massage satisfaction.

The duration is the average time spent for each massage session. According to a past study, short-term and long-term benefits may be attributed to the duration [19]. Either of the two is a potential outcome after experiencing massage because the service aims to alleviate pain. However, massage should only be performed at a recommended duration because relief can become a pain if time exceeds significantly [20]. Otherwise, a massage performed at a very short duration might be ineffective. On one hand, a study supported that short duration for a couple of massage types can increase the social bond cognition of consumers [21]. They tend to build a good rapport with massage therapists, peers, and colleagues after a short duration of massage is performed. Another research seconded the inclusion of duration as an important factor in massage technique because duration should complement the overall massage needs of a consumer [22]. Thus, it is important to identify the right amount of time preferred by massage parlor consumers.

The type of room refers to spatial size alongside the number of massage consumers it can accommodate. Some researchers performed experimental massage studies in a single room but categorized participants into three different groups [23]. Despite the presence of only one type of room, they noticed the effectiveness of massage in minimizing the pain of participants from two groups [23]. Meanwhile, a semi-large room was utilized by other researchers but ensured that the patient:bed ratio was 1:1 [24]. They showcased that experimental studies could still be performed successfully despite the presence of a large space as long as experimental subjects are comfortable and well-informed about the research objective [24]. Interestingly, some scholars decided to evaluate both small space and large space rooms [25]. The small space caters to individual massage customers while the large space can accommodate multiple consumers. All these existing studies deliberated the criticality of choosing the right type of room dependent on the experimental objective.

Next, the massage type as seen in the framework implies the specific massage that a consumer would avail from the clinic. It can either be a recommendation from a massage professional if consumers suffer from an aggravated health issue or their personal choice if health issues are not a concern. For instance, researchers tested different massage types for postpartum mothers [7]. They supported that common massage types supported by other studies are still preferred by 8 out of 10 postpartum mothers [7]. Their findings supported the importance of testing at least three types of massage and hearing opinions from the subjects. Another scholarly work noted that the administration of massage services, depending on the type of massage, plays a significant role in balancing the overall satisfaction of consumers [26]. This occurs because the massage therapist’s technique, massage parlor’s environment, and tools (e.g., lotion, oil) vary.

Google review rating is the last attribute displayed in the framework. It is an online evaluation posted voluntarily by consumers. Consumers have the option to post feedback in qualitative and quantitative form. These online reviews help future consumers decide whether to purchase a service or not [15]. Consumers tend to instill trust in online reviews because they share the same intention to avail of a product or service [17]. Through Google reviews, customer preferences are determined easily and stakeholders can improve their services accurately [27]. Some consumers are hesitant to provide feedback on the spot but most appear vulnerable to their honest opinions once they utilize Google reviews.

## Methodology

This study was approved Mapua University Research Ethics Committees (FM-RC-22-06-06) and was conducted from September 2022 until November 2022. All participants were asked to fill out an online consent form before the data collection. In addition, all respondents were also informed of the details of the questionnaire and the purpose of the study.

### Respondents

Respondents were gathered through a convenience sampling approach where the questionnaire was distributed to random people and at random times. Convenience sampling was the best technique since the conjoint approach maximized experimental research by investigating stimuli that were never explored by other studies. These stimuli were also nonrepeating and respondents’ brains were stimulated continuously, ensuring that data was accurate.

Furthermore, the questionnaire was hosted by Google Forms and distributed to Indonesians on various social media platforms, such as Facebook, Instagram, and Twitter. According to a past study, these are top social media platforms that were popular among consumers globally and were often used by researchers in the research setting [28]. These aforementioned platforms contain Indonesian-related social groups where researchers joined and distributed the questionnaire. Moreover, the researchers maximized their current network and sent the questionnaire to their connections, such as relatives and friends. They were given a link to the Google Forms and instructions before filling out the form. All participants willingly participated in the study by signing the consent form. Apart from agreeing to participate in the study, the objective of the questionnaire was also indicated. And since the participants would answer the questionnaire in an online setting, the researchers did not induce any time limits. This also guaranteed that participants would complete the questionnaire voluntarily at their own pace and without time pressure. After respondents filled out the consent form, the researchers provided filter questions to ensure that the respondents would best describe the needed sample size of consumers in a massage parlor. First, the respondents were asked if they were Indonesians living in Indonesia currently. Otherwise, their responses were excluded. Second, respondents were asked about their number of massage experiences. Those who answered zero were excluded from the final number of respondents.

The sample size was adequate by applying the formula presented by Sun et al. [11]:

$$\frac{500c}{\left(t\right)\left(a\right)}$$ (1)

where *c* is equivalent to 6 attributes, *t* is equivalent to 27 tasks or stimuli, and *a* is equivalent to 1 alternative, resulting in 112 minimum valid respondents. As a result, the researchers gathered a total of 212 valid respondents.

Based on Table 1, less than half (38.2%) of the respondents were male, and more than half (61.8%) were female. In addition, the respondents were between 18 to 61 years old (average: 25.89 years old) and were mostly dominated by 18 to 24 years old. The occupation was dominated by full-time employees at 56.1%, followed by students at 37.3%, and housewives/househusbands at 2.4%. The frequency of visiting a massage parlor in a month is not significantly different. Nevertheless, the highest was 31.6% whereby respondents visit massage parlors less than once a month and the lowest was 19.3% capped with more than four visits a month. Interestingly, the distribution of the price preferences is almost uniform for the first three classifications. However, they had a notable percentage decrease for the highest amount (IDR 250,000) since only 9% of respondents had the willingness to pay the most expensive price.

**Table 1:** The demographics of the respondents.

Demographic	Classification	Frequency	Percentage
Gender	Female	131	61.8%
	Male	81	38.2%
Age	18 to 24 years old	113	53.3%
	25 to 34 years old	85	40.1%
	35 to 44 years old	6	2.8%
	45 to 54 years old	6	2.8%
	At least 55 years old	2	0.9%
Occupation	Employee	119	56.1%
	Student	79	37.3%
	Housewife/househusband	5	2.4%
	Entrepreneur	3	1.4%
	Others	6	2.8%
Massage house visitation frequency	Less than once a month	67	31.6%
	1 to 2 times a month	47	22.2%
	3 to 4 times a month	57	26.9%
	More than 4 times a month	41	19.3%
Average cost of every visit	IDR 100,000 to 150,000	63	29.7%
	($6.3-$9.5)		
	IDR 151,000 to 200,000	67	31.6%
	($9.6-$12.7)		
	IDR 201,000 to 250,000	63	29.7%
	($12.8-$15.9)		
	More than IDR 250,000	19	9%
	(More than $15.9)		

### Conjoint design

Table 2 displays the selected attributes and corresponding levels that affect consumers’ preferences for choosing a massage parlor. This reflects the orthogonal design of the conjoint analysis, which is the beginning of the conjoint process. The questionnaire distributed to participants was derived from a combination of these attributes and levels. Specifically, six attributes were evaluated in this study, such as gender of the massage therapist (male, female, no preference), price of massage every session (IDR 100k–150k, IDR 150k–200k, IDR 200k–250k, > IDR 250k), duration of each massage session (60 mins, 90 mins, 120 mins), type of massage room (solo: 1 bed, couple: 2 beds, common room), massage type (oil massage, reflexology, acupuncture and cupping, oil massage, and hot stone), and Google review rating (> 4.6, 4.6–4.3, 4.3–4.0, < 4.0).

**Table 2:** The attributes and levels for massage parlors.

Attributes	Levels
Gender of Massage Therapist	Male, Female, No Preference
Price	IDR 100k-150k, IDR 150k-200k, IDR 200k-250k, > IDR 250k
Massage Duration	60 mins, 90 mins, 120 mins
Type of Room	Solo (1 bed), Couple (2 beds), Common Room
Type of Massage	Oil Massage, Reflexology, Acupuncture and Cupping, Oil Massage and Hot Stone
Google Review Rating	> 4.6, 4.3–4.6, 4.0–4.3, < 4.0

First, the gender of the massage therapist could be an important consideration for most consumers because massage entails physical contact. Thus, the researchers evaluated male massage therapists, female massage therapists, and no gender preferences. In Iran, Ghasemi et al. [10] conducted a study using female massage therapists to analyze the effects of massage type on female patients with leg concerns. Similarly, in Germany, the same gender issue between the athlete and massage therapist was investigated by Reichert [18]. In this past study, male patients treated by female therapists showed an elevated mood increase. Female massage therapists were in demand in both studies regardless of the consumers’ genders [10, 18]. While it was a common practice to choose female over male massage therapists, a study recommended investigating the importance of male massage therapists since massage parlors were prohibited from focusing on one gender hiring process [3]. Meanwhile, another study noted that consumers without massage experience did not mind the gender of massage therapists [4].

Second, the massage treatment price was an essential factor in the consumer’s decision-making process because financial resources mattered to the majority of consumers. In Indonesia, the average cost of a 60-minute-long full-body oil massage is IDR 75,000, reflexology costs around IDR 65,000, partial acupuncture is approximately IDR 120,000, and hot sone averages IDR 100,000. The study used these values as a benchmark to evaluate the following price level intervals: IDR 100k-150k, IDR 150k-200k, IDR 200k-250k, > IDR 250k. It was discovered that massage price was a significant and positive factor influencing consumer satisfaction [3, 4]. This implied that the economic situation of consumers would force them to choose cheap options while others would not have any issues in choosing expensive costs.

Third, the duration of the massage could increase the massage parlor’s attractiveness because the time sessions were equated to relaxation. Also, it was often paired with price as people often looked to get their money’s worth by factoring in the time spent. The most common time allowances serviced are 60 minutes, 90 minutes, and 120 minutes. These time limits were dependent on the type of relaxation the consumer wanted. More importantly, a decrease in pain could be achieved with appropriate time intervals and frequency of visits [19]. But then, both consumers and massage parlors could have a tight schedule; thus, less time and visits might be beneficial to some.

Fourth, consumers have the power to choose from the available rooms. In the usual experimentation setup, massage respondents were placed in a single room [23]. Some preferred to be in a separate solo room due to privacy. This setup coincided with the evaluated levels as the present study would also like to determine the significance of a room that consists of a single bed. Moreover, it was seen that couples who visited a massage parlor wanted to share a room. Couples who engaged in massage were more relaxed as they gained companionship [1]. This result showed that consumers would relate to each other easily if they could share the same massage service experience with another person because of the feeling of belongingness. In addition, the inclusion of a common room was another hypothetical preference. Consumers who lacked experience were deemed fine with any kind of massage services, including the type of room [4].

Next, the type of massage was investigated because each type is specialized based on the person’s needs. This study assessed the common massage services, namely oil massage, reflexology, acupuncture and cupping, and oil massage coupled with hot stone. The oil massage was recommended by pharmacology experts since the therapists infuse essential oil on the customer’s body with mild pressure [10]. Reflexology is a foot massage that targets reflex points such as hypothesis, thyroid, parathyroid, pancreas, adrenal glands, and solar plexus [10]. Meanwhile, acupuncture utilizes thin needles and cupping employs cups. Both of these target a specific area that helps relieve swelling and pain [29]. Other consumers preferred oil massage and hot stone combination because heated basalt stones are placed on the patient’s chakra points [30].

Lastly, Google review ratings supplied the world with accessible information about massage parlor services. They reflected the metrics based on the past experiences of consumers. The present study categorized Google review ratings as follows: > 4.6, 4.6–4.3, 4.3–4.0, < 4.0. They were compartmentalized based on the actual data distribution. A past study noted that Google review rating was the most convenient platform because it was time-saving and user-friendly [31]. Users also used this platform frequently for solo and family leisure activities, like massage services [31]. The degree to which people gave credit to the reviews shown on Google varied among potential customers. Two different scenarios might occur as some customers could be influenced by online ratings easily while others could prefer trying the massage parlor’s services.

### Conjoint analysis

The conjoint analysis was performed through IBM’s statistical analysis software, Statistical Package for the Social Sciences (SPSS). This analysis was performed to gauge the preferences of massage parlor consumers. It was perceived to be one of the most effective tools in consumer behavior and market research [5]. For instance, a past study used conjoint analysis to improve employee welfare, which made employees more productive and effective at work [12]. This also resulted in profit returns as employees hit more metrics. Hence, many business stakeholders employ this technique to improve their operations. The conjoint analysis could be performed by selecting attributes and levels from relevant studies, and ultimately, combining the best elements [11]. Attributes represent the service’s or product’s features while levels are the corresponding sub-characteristics of attributes. It was deemed necessary to choose only the most relevant attributes and levels to ensure the questionnaire’s reliability [12]. Since conjoint analysis was also considered a multivariable tool, weights of attributes and levels were determined [12]. Therefore, their corresponding values would best represent the perceptions of consumers.

Since conjoint analysis took the form of a multiple regression model, its equation is described as follows:

$$y=\;a_{0}+a_{1}x_{1}+a_{2}x_{2}+a_{3}x_{3}+a_{n}x_{n}+e$$ (2)

where *a* refers to the parameters of the model, *x* pertains to values of attributes, *n* is the number of attributes, and *e* means random variable. Expert ratings were then applied to calculate the possible values of each *x*_*n*_ resulting in varying *y* values.

Afterward, the ranking of data was calculated as:

$$p_{ki}^{*}=\left(k+1\right)-p_{ki}$$ (3)

where *k* is the stimulus, *i* is the respondents, and *p* is the ranking of data. The ranks were transformed using averaging techniques for each rating estimate. Thus, the respondents’ preferences were discovered through attribute level’s ranking, importance, and utility values.

**Fig 2:**
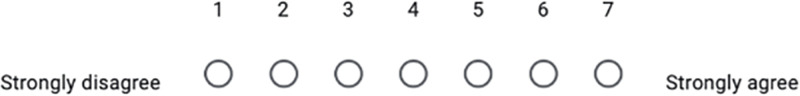
Illustration of 7-point Likert scale.

A total of 27 stimuli (Table 3) were generated through an orthogonal design with 6 attributes. The researchers tested 2 holdouts for data consistency [5]. During the collection, a 7-point Likert scale was utilized to evaluate the respondents’ preferences. 7-point Likert scale is a semantic differential scale that gauges the preferences of respondents. Whereas, 1 represents “Strongly not preferred” and 7 represents “Strongly preferred”. Respondents could select the magnitude value of their dislikeness or likeness for each stimuli combination from 1 to 7. Each stimuli combination from Table 3 was presented one at a time to respondents. For example, the first item would show the following combination: “Gender of massage therapist: Female, Price: IDR 200k-250k, Massage Duration: 60 mins, Type of Room: Couple (2 beds), Type of Massage: Reflexology, Google Review Rating: < 4.0”. Below each item, participants were asked the following question: “Kindly rate the following combination of massage parlor services based on your preferences:” whilst attaching Fig 2 in the questionnaire.

**Table 3:** Twenty-seven stimuli of the conjoint design.

Combination	Gender of Massage Therapist	Price	Massage Duration	Type of Room	Type of Massage	Google Review Rating
1	Female	IDR 200k-250k	60 mins	Couple (2 beds)	Reflexology	< 4.0
2	Male	> IDR 250k	90 mins	Solo (1 bed)	Acupuncture and Cupping	< 4.0
3	Female	> IDR 250k	60 mins	Common Room	Oil Massage and Hot Stone	> 4.6
4	Female	IDR 100k-150k	90 mins	Solo (1 bed)	Oil Massage	4.3–4.0
5	Female	IDR 100k-150k	60 mins	Solo (1 bed)	Reflexology	> 4.6
6	Male	IDR 100k-150k	120 mins	Couple (2 beds)	Oil Massage and Hot Stone	< 4.0
7	Male	IDR 150k-200k	60 mins	Solo (1 bed)	Oil Massage and Hot Stone	> 4.6
8	Female	IDR 150k-200k	90 mins	Common Room	Oil Massage	< 4.0
9	Female	IDR 100k-150k	90 mins	Couple (2 beds)	Oil Massage and Hot Stone	4.6–4.3
10	Female	IDR 100k-150k	90 mins	Couple (2 beds)	Acupuncture and Cupping	> 4.6
11	No preferences	IDR 150k-200k	90 mins	Couple (2 beds)	Reflexology	> 4.6
12	Male	> IDR 250k	90 mins	Solo (1 bed)	Reflexology	4.6–4.3
13	Female	IDR 100k-150k	120 mins	Solo (1 bed)	Oil Massage	> 4.6
14	Male	IDR 200k-250k	60 mins	Couple (2 beds)	Oil Massage	4.6–4.3
15	No preferences	IDR 200k-250k	90 mins	Solo (1 bed)	Oil Massage and Hot Stone	4.3–4.0
16	Male	IDR 100k-150k	60 mins	Solo (1 bed)	Oil Massage	> 4.6
17	Male	IDR 100k-150k	90 mins	Couple (2 beds)	Oil Massage	> 4.6
18	No preferences	IDR 100k-150k	60 mins	Common Room	Acupuncture and Cupping	4.6–4.3
19	Male	IDR 100k-150k	120 mins	Common Room	Reflexology	4.3–4.0
20	Female	IDR 200k-250k	120 mins	Solo (1 bed)	Acupuncture and Cupping	> 4.6
21	Male	IDR 150k-200k	60 mins	Couple (2 beds)	Acupuncture and Cupping	4.3–4.0
22	No preferences	> IDR 250k	120 mins	Couple (2 beds)	Oil Massage	> 4.6
23	Female	> IDR 250k	60 mins	Couple (2 beds)	Oil Massage	4.3–4.0
24	Female	IDR 150k-200k	120 mins	Solo (1 bed)	Oil Massage	4.6–4.3
25	Male	IDR 200k-250k	90 mins	Common Room	Oil Massage	> 4.6
26	No preferences	IDR 100k-150k	60 mins	Couple (2 beds)	Oil Massage and Hot Stone	> 4.6
27	No preferences	IDR 100k-150k	60 mins	Solo (1 bed)	Oil Massage	< 4.0

## Results

Table 4 represents the utility estimates, standard error of each level, and average importance values of attributes. The utility estimates for each corresponding attribute level were totaled to zero, signifying that levels were ranked appropriately. For gender attribute levels, the female massage therapist was preferred, next to no preference, and the male. Meanwhile, the price of massage was favored as arranged in this order: IDR 100k-150k, IDR 150k-200k, > IDR 250k, and IDR 200k-250k. Then, consumers preferred 120 minutes massage duration over 90 minutes and 60 minutes. Surprisingly, they commonly chose the common room, then a couple room, and the least chosen was the solo room. For the type of massage, reflexology was highly favored, with a 2^nd^ priority in acupuncture and cupping, third priority was oil massage, and the last priority was oil massage and hot stone. Finally, a greater than 4.6 Google review rating held the highest utility estimate, the 4.3–4.6 range topped second, 4.0–4.3 got the third rank, and less than 4.0 was the last one. Meanwhile, attributes’ average importance values were arranged from highest to lowest as follows. Google review rating was found to be the most significant attribute (68.5%), followed by gender of massage therapists (12.4%), massage type (9.6%), room type (4.5%), duration (3.6%), and price (1.4%).

**Table 4:** Utility estimates and average importance values.

Attribute	Level	Utility Estimate	Standard Error	Average Importance Value (%)
Gender of Massage Therapist	Male	-0.184	0.067	12.4
	Female	0.17	0.067	
	No Preference	0.014	0.08	
Price	IDR 100k-150k	0.022	0.074	1.4
	IDR 150k-200k	0.005	0.091	
	IDR 200k-250k	-0.017	0.091	
	> IDR 250k	-0.01	0.091	
Massage Duration	60 mins	-0.043	0.067	3.6
	90 mins	-0.018	0.067	
	120 mins	0.061	0.08	
Type of Room	Solo	-0.073	0.067	4.5
	Couple	0.018	0.067	
	Common Room	0.055	0.08	
Type of Massage	Oil Massage	-0.016	0.074	9.6
	Reflexology	0.119	0.091	
	Acupuncture and Cupping	0.051	0.091	
	Oil Massage and Hot Stone	-0.154	0.091	
Google Review Rating	> 4.6	0.606	0.074	68.5
	4.3–4.6	0.502	0.091	
	4.0–4.3	0.238	0.091	
	< 4.0	-1.346	0.091	
(Constant)		4.881	0.061	

After the calculation of utility estimates, the stimuli’s corresponding utility estimates for each attribute level were computed through summation. Table 5 indicates the ranking of 27 evaluated stimuli. The top three stimuli were combination numbers 10, 5, and 20, respectively. To highlight the stimulus that ranked first, combination number 10 comprised the following levels: female massage therapist, IDR 100k-150k price every session, 90 minutes massage duration, couple room with two beds, acupuncture and cupping massage type, and massage parlors with greater than 4.6 Google review rating.

**Table 5:** Ranking of each stimulus.

Stimuli Combination	Utility Estimate Total	Rank
1	-1.099	23
2	-1.58	26
3	0.624	8
4	0.323	16
5	0.801	2
6	-1.583	27
7	0.157	20
8	-1.15	24
9	0.54	10
10	0.849	1
11	0.744	5
12	0.336	15
13	0.77	4
14	0.26	19
15	-0.01	22
16	0.312	17
17	0.428	12
18	0.601	9
19	0.311	18
20	0.798	3
21	0.085	21
22	0.673	6
23	0.357	14
24	0.649	7
25	0.426	13
26	0.463	11
27	-1.442	25

Table 6 demonstrates the necessary statistical values that support the study’s conjoint design. Pearson’s correlation coefficient yielded 0.983 with a 0.001 significance level. These values were acceptable as the required parameter was at least 0.70 [32]. Thus, the factors in conjoint design had significant and strong positive relationships. Furthermore, the yielded Kendall’s Tau was 0.809 with a corresponding significance value of 0.001. Based on credible research, the minimum value for Kendall’s Tau was 0.70 [32]. Hence, the presented orthogonal design was satisfactory. Finally, a 1.00 Kendall’s Tau for holdouts implied data consistency as presented in the current study and supported by Ong et al. [5].

**Table 6:** Correlation and Kendall’s Tau results.

Statistical Analysis	Value	Significance
Pearson’s R	0.983	0.001
Kendall’s Tau	0.809	0.001
Kendall’s Tau for holdouts	1.000	

## Discussion

### Interpretation of conjoint results

Google review rating was found to be the most important attribute for massage parlors. The difference in importance between Google reviews and the other attributes was significant as it yielded 68.5% preference. Hence, the remaining 31.5% were distributed among the remaining five attributes. It was discovered that massage parlors that had high reviews were consistently favored by customers of all backgrounds. The higher the value of the Google review, the more popular the parlor was. Specifically, massage parlors with a Google review higher than 4.6 stars out of 5 stars garnered the most positive responses. Google applies a certain algorithm to calculate star reviews. Although some users can give a very bad review with a very poor rating, all the other reviews will also be taken into account in the standardized calculation. Hence, the final rating was still deemed accurate and would best represent the online reviewers’ feedback. Moreover, Google has a built-in feature that allows users to see the most legitimate reviews by considering the impact of the overall ratings with the corresponding stars.

For instance, a hundred thousand Google reviews from thousands of restaurants underwent a sentiment analysis algorithm [27]. This past study discovered that restaurants could focus on service quality if services they received 1 star, food, and atmosphere for 2 stars and 3 stars, food improvement for 4 stars, and price for 1, 2, and 3 stars. While the current study limited itself to finding important attributes, it presented a starting point to expound on factors affecting Google review ratings. El-Said [33] indicated that positive star reviews had good feedback but had little effect on booking intentions, however, those with negative reviews affected businesses greatly. But the current study argued that both positive and negative reviews impacted the massage parlor’s operations. In another study, Weisstein et al. [34] revealed that consumers with purchase goals were more negatively affected by the number of negative reviews than consumers without purchase goals. The current study evaluated consumers with a purchase goal, which was to enjoy the services of a massage parlor. Thus, the primary reason for incurring a negative utility estimate for the least Google review rating. Connecting positive and negative feedback to another study, researchers concluded that online reviews aid consumers in finalizing their market purchases [15]. The term “online reviews” topped the data mining keyword extraction across five research areas published for more than ten years [15]. This further supported Google Review’s effectiveness as an attribute of massage service consumers. Another research utilized online reviews to generate a product that best described consumers’ needs [35]. Although the previous study utilized a product concept, similarities between the product and service industry were both visible as the present study was also focused on meeting customers’ requirements. This implication proved that stars from Google reviews were directly associated with consumers’ level of satisfaction.

The gender of massage therapists was found as the second most important attribute (12.4%) considered by the consumers. The vast majority of customers favored a female therapist over a male one. The difference in the preference was significant, too. Female therapist’s utility estimate garnered a positive value compared to male therapist’s utility with a negative value. On a similar note, Baskwill and Vanstone [3] concluded that female therapists were preferred to men due to comfortability concerns. Negative connotations surrounded male therapists treating the opposite gender due to social norms. This was highly evident in the present study as more than half of the respondents were female and felt more relaxed being treated by female therapists. Another probable reason was that female therapists dominated the massage industry in many countries, not only in Indonesia. This reason was supported by Kruger et al. [36] as they concluded that South Africa’s female therapists were prone to osteoarthritic symptoms due to the high demand for their services from consumers. In Switzerland, female therapists were greatly satisfied with their job, being of service to their consumers, and some would offer massage services as freelancers [20]. Freelance massage therapists were also common in Indonesia and some businesses would hire part-time employees depending on the surge in demand.

Surprisingly, the current findings also showed that no gender preference was secondly favored with a positive utility estimate and still dominated male therapist attribute level. A similar study discovered that both genders reeived positive massage feedback from male and female consumers, which undermined the preference for male massage therapists [7]. Likewise, Reichert [18] mentioned that the gender of the therapist had no significant impact on the psychological health of the patient except for male athletes being treated by female therapists. They supported the secondary findings of the present research where no gender preferences could also be an option if female massage therapists were unavailable. Result differences still occurred because this was only the second optimal solution for the present study. It happened because the past study focused on sports athletes while the current study maximized general consumers’ perceptions. Meanwhile, another study expressed good feedback on no gender preferences if participants were first-time visitors of the massage parlor [4]. This scenario posited that repeat customers were more conscious, resulting in a proactive selection of a specific massage therapist. In comparison to the current study, researchers considered consumer visit frequency, and the numbers were generally balanced. Thus, the current study argued that the lack of gender preference factor was not associated with massage parlor visits. However, there was evidence of controlling the group of respondents instead of the gender of the massage therapist [10]. They noted that female consumers tend to have a less biased finding compared to male consumers [10]. The current study argued that the gender of consumers should not be controlled because it would restrict research findings.

Massage type was found to be the third most important attribute (9.6%) in affecting the desirability of a massage parlor. Nik Yusof Fuad et al. [7] investigated different massage types and identified that consumers tended to prioritize common massage techniques applicable to their current condition. They settled for commonalities because the subjects were postpartum mothers who needed effective massage services and not for experimentation purposes. Unlike the current study where subjects consider massage services for either health or relaxation purposes. Li et al. [21] supported that the massage type could bring different kinds of pleasure due to an increase in oxytocin that influences social bonds and cognition. Both Li et al. [21] and the present study gave importance to massage types that utilize therapists’ hands. It was identified that massage types that require body pressure were better than the types that require machines. In another study, Margenfeld et al. [37] experimented with massage type modification and found that the modified type was more effective in their subjects compared to the common massage offered to patients. The past study was aligned with the importance of massage type since it was deemed necessary to explore massage types that best suit patients’ preferences and needs. Likewise, Kennedy et al. [38] noted that choosing a specific treatment was a crucial factor because therapists must ensure that the right massage type was offered to patients. The present study had a similar discernment since therapists assess their patients. Hence, an appropriate diagnosis would be a good indicator for consumers. Otherwise, consumers who availed of massage types that could not address their health concerns would affect the massage parlors negatively.

Among the four evaluated massage types, reflexology was mostly preferred by customers, followed by acupuncture and cupping. The oil massage was found to be far less significant in affecting the attractiveness of a massage parlor. Oil massage paired with hot stones combination was the least attractive to customers and the least likely to be picked among all massage types. These results were aligned with the research done by Ashley J. Farrar and Francisca C. Farrar [26], which stated the rising popularity of reflexology. While there were minimal studies on Indonesians’ massage preferences, the current findings concluded that reflexology was favored by most of the sample population. However, reflexology was found the least common massage, and hot stone massage topped the massage type in a study by Nik Yusof Fuad et al. [7]. This occurred because the past study investigated massage effects on postpartum women while the present study did not assess a specific condition of patients. Hence, the current study represented the preferences of consumers who did not need immediate medical attention. On one hand, table and chair massage focusing on the consumer’s focal point was prioritized by Smith et al. [13]. Their massage types were centered on relaxation treatments because they also provided post-treatment service. Although the previous study did not utilize any massage types similar to the current research, the present study was more conclusive in finding multiple attributes influencing good massage experience.

Room type was the fourth most important factor (4.5%) preferred by customers. Interestingly, results showed that consumers delivered positive feedback on shared rooms. Specifically, consumers favored the common room the most, and next was the couple room with two beds. These findings matched the study of Naruse & Moss [1], whereby massage parlor consumers could relieve more stress when they felt a sense of belongingness through the people who availed of the same services. On a similar note, staying in semi-private rooms that could accommodate 48 patients was not a deal breaker for patients [24]. Regardless of the setting, may it be the hospital or massage parlor, patients did not mind undergoing massage therapies using a common room. Although Dehcheshmeh & Rafiei [23] placed control groups in a single room and generated significant massage intervention results, the present study contended that solo rooms were highly unfavored by consumers. Despite the convenience and safety advantages of solo rooms, they did not garner attention due to their expensive cost compared to shared rooms that were more economically accepted by regular consumers. In an experimental study performed by Ashton et al. [25], massage therapies were done in dressing rooms, changing rooms, and waiting areas while patients were waiting to be accommodated in a private room. The present study argued that the past study’s experimental setup was deemed inconvenient. Although participants could benefit from the pre-massage offered by the company, the business could suffer since they needed to hire employees who could assist people in waiting lines. Limited solo rooms and long waiting lines were considered huge disadvantages of solo rooms in massage parlors, thus consumers of this study did not favor them. In a different experimental context, massage therapies were performed using the classroom setting since researchers were medicine-allied students [39]. Despite the unconventional setup, a hospital setup was made almost similar, resulting in the improvement of massage therapy techniques. The previous study demonstrated that room type, including the ambiance and layout, could be modified based on consumer’s needs.

The duration of the massage ranked second to the last with only 3.6% importance value. The investigation durations ranged around 60 minutes, 90 minutes, and 120 minutes. Based on the results, longer massage duration (120 mins) was positively accepted while shorter massage durations (90 minutes and 60 minutes, arranged respectively) were negatively accepted. Similarly, Li et al. [21] guarantee that higher massage visits and greater massage durations generated positive outcomes for patients. Human bodies need time to absorb massage benefits, thus 120 minutes was considered the optimal massage duration. However, most researchers used ≤ 60 minutes of massage duration for their experimental studies [19, 24]. Thus, the present study recommended increasing the massage duration to future scholars as it would help increase the reliability of investigational data. In contrast to the study of Barth et al. [20], they revealed that 60 and 90 minutes were enough to address patients’ health concerns. However, the previous study focused on the experimental aspect and also failed to incorporate a better statistical technique. On the contrary, massage duration could be the most important attribute if researchers were to focus on laboring mothers. For example, Bolbol-Haghighi et al. [40] exhibited the criticality of duration based on labor stages because the right amount of time spent for each labor should vary. Laboring mothers was more sensitive, thus, medical professionals should pay more attention to massage therapy’s duration. Unlike the current study that did not include severe medical cases, present research allowed leeway for consumer’s purpose of availing of a massage service.

Surprisingly, the price of massage every session was found to be the least important attribute with a 1.4% importance score. This meant that customers prioritized other attributes than massage price and were more than willing to pay a relatively higher cost to have a better experience. The prices ranged from 100k to 150k, 150k to 200k, 200k to 250k, and more than 250k in IDR currency. These ranges were considered reasonable based on Indonesia’s cost of living. In Canada, one massage therapy could cost 240 CAD (approximately 2.7M IDR); meanwhile, regular acupuncture in the U.S. amounted to 352 USD (approximately 5.3M IDR) [41]. The price gap among those countries was huge because geographical location played a crucial role in this part. Indonesia was budget-friendly compared to Canada and the U.S. Although the least amount still garnered positive feedback from consumers, the difference in price preferences was considered insignificant due to the proximity among utility estimate values. Similarly, high-income earners would not mind paying for a hefty sum unlike low-income earners who would choose wisely before purchasing a service [16]. This implies that consumers control their purchasing behavior due to limited resources and the spending behavior depends on one’s income capability. Likewise, Lee and Zhao [42] believed that customer convenience represented the cost value, leading to consumer retention. Both the present and past studies discussed that the primary services dominated consumers’ preferences instead of the price Furthermore, a study discovered that cost only had an indirect effect on customers’ perceptions of the service industry [43]. The indirect connection held lesser importance than a direct relationship, which was another likely reason for placing the price at the lowest attribute importance. The participants of the study perceived that price should be the least factor they should be wary of when availing packages at a certain massage parlor. Coinciding with the study of Kumar et al. [44], the cost did not equate to effectiveness. Most likely, patients would prefer seeing the actual result after the session. They focus more on availing of services from credible massage parlors with great service packages coupled with reliable therapists.

### Managerial implications

This paper aimed to contribute to the massage industry, especially in Indonesia. Conjoint analysis was utilized to determine the important attributes. This research also showcased the levels of consumer preferences influencing the massage parlor’s business operations. Overall, the best combination that must be maintained by stakeholders was the inclusion of a female massage therapist, IDR 100k-150k massage price every session, 90 minutes massage duration, couple room with two beds, acupuncture and cupping massage type, and massage parlors with greater than 4.6 Google review rating. If massage parlors were to maintain these services, they could maintain the competitiveness of the business and even enhance future business developments.

Meanwhile, massage parlors could also focus on employing strategies based on the importance values of each attribute and their corresponding levels. Out of the 6 factors, Google review rating was found to be the most significant attribute (68.5%), followed by gender of massage therapists (12.4%), type of massage (9.6%), and other attributes. These top three attributes must be prioritized by massage parlors.

Most importantly, it was suggested to maintain more than a 4.6 Google review rating as this was deemed the most crucial factor while expanding the industry. Google review ratings were garnered based on the customer’s overall customer experience during their massage session. Business owners should focus on giving the best experience starting with a well-trained massage therapist, clean and hygienic rooms, and even the smallest details such as oil selection. In terms of marketing, some strategies could be utilized such as pre-determining consumers who seemed to be enjoying the services and encouraging them to post positive feedback and give five points rating on Google review. An alternative approach would be providing huge discounts or free services to random Google review raters. If budget would permit, it could also be a good approach to give promotional discounts to each customer who would write positive comments and give a five-star rating about their massage experience. These approachers were assumed to encourage customers to leave an optimistic review that would also help the business grow. Subsequently, the local government should evaluate the massage industry in their regions. For instance, massage parlors could offer free services to regional government officials. These officials could endorse promising regional massage parlors through digital platforms, including Google review ratings. This specific government-business connection would ignite the trust of future customers to visit a specific massage parlor. Furthermore, the business owners were encouraged to respond to the customer’s review rating professionally regardless of the customers’ positive and negative insights. By doing so, a well-structured communication image and receptive track record could be seen by potential future customers. Generally, massage parlor stakeholders must maximize the abovementioned suggestions to help them grow their business digitally as Google review ratings impact consumer influences exponentially.

Additionally, business owners must be wary of the massage therapists’ that they would hire. Employing a higher number of females compared to male massage therapists would produce a better outcome since most customers preferred female massage therapists. Nonetheless, a massage parlor should not forget to hire at least one male therapist since some consumers did not mind gender preferences. This approach also ensured that gender discrimination would be eliminated since this factor might affect the perceptions of other customers. To eliminate profit loss, a part-time or on-call male therapist could be a good option to ensure that labor expenses were minimized. In terms of the massage therapist ratio, massage parlor stakeholders could also opt for 80% female and 20% male. These recommendations could allow massage parlors to handle more customers who preferred to have female therapists. They could also mitigate the risk of lost sales due to the lack of female massage therapists.

Finally, the massage type offered by massage parlors held an important value as it ranked third among the six attributes. Massage parlors in Indonesia were encouraged to focus on reflexology and acupuncture with cupping. While it was still okay to offer other massage options to customers, the researchers encouraged business owners to have their massage therapists training. Specifically, they recommend in-depth training about reflexology and acupuncture with cupping. The management should also impose certification and diploma courses relevant to these massage types. These approaches would assure customers that professionals assigned to them have a mastery of skills. Moreover, the management should focus more on finding the best oil, needles, cups, and other products needed to perform reflexology and acupuncture with cupping. These strategies were expected to produce good service to customers. They would feel safe in the hands of professional massage therapists and massage parlors with well-equipped equipment.

### Theoretical implications

The current study’s stimuli could be considered the primary benchmark for expanding massage parlor services. It was previously discussed in the literature review that none of the past studies explored the evaluated attributes and levels in understanding consumers’ preferences for choosing a massage parlor. More importantly, findings revealed the ranking of each attribute and the importance of each attribute’s levels. In addition, it could be depicted that Indonesia’s massage services were rarely explored in the academe setting. The present study employed a quantitative approach to ensure that data is maximized and interpreted accurately.

It was declared by other studies [3, 4] that consumers gauge their loyalty and purchase services based on massage services. Thus, the researchers’ statistical approach provided a new perspective to business stakeholders. Specifically, the conjoint analysis was utilized to analyze 27 stimuli. These stimuli underwent an orthogonal design and found the best combination. This best combination could be utilized theoretically to form other types of stimuli that best fit the massage industry. Therefore, the conjoint’s orthogonal design delivered a new perception of prioritizing both consumers and the business side as some would only focus on one or the other.

### Limitations and future research

Although the researchers accomplished the necessary objective, they acknowledged limitations that could enhance the study findings. The first limitation was the imbalanced frequency among the demographic characteristics of participants. Since the questionnaire was mostly answered by the younger generation, the researchers gathered mostly from the age range of 18 to 34 years old. Other studies could create a comparison between the responses of students considering that customers of at least 35 years of age could have a different opinion than the younger generations. It is recommended to distribute the questionnaire on-site and other scholars could also opt to form a partnership with corporate, NGO, and government agencies to cover a variety of demographics. With this strategy, non-probability sampling techniques could also be applied to address biases and generalizability issues. In line with this, future participants may be asked health-related questions once the age range is neutralized. The researchers perceived that current participants would not inflict severe health conditions since the younger generation was deemed healthier than the older ones. Thus, future scholars could identify a different perception once both generations with few and severe health conditions were evaluated. Despite these limitations, the present study discovered interesting findings about customers’ favored perceptions which were not discovered by existing studies.

Secondly, other scholars may choose to expand the methodology by adding data analytics methods. Specifically, machine learning and metaheuristics approach to cluster customers accurately and model exploration could be considered in the study’s expansion. For instance, k-means and particle swarm optimization can be combined to cluster massage parlor consumers. The Ward’s method can also be merged with K-means to further enhance cluster accuracy. However, one drawback of clustering through these aforementioned approaches is the customization of algorithm parameters more than the factors affecting consumer behavior. Thus, before finding accurate market segmentation, future scholars are encouraged to test more attributes with customized parameters. Meanwhile, the researchers achieved their primary objective of finding massage parlor consumer preferences without adjusting any parameters because the conjoint analysis can stand on its own, which is the biggest highlight of the study. In addition, since the researchers found Google review rating was the most important attribute, future researchers could extract real-time data from Google and find more implications that further describe the customer’s feedback. They could also propose factors that could be improved to increase massage parlors’ Google review ratings. Nevertheless, the current study discovered the most important attribute and appropriate market strategy.

The last limitation of the study was the exclusion of the massage parlor’s specific service characteristics. For instance, facilities cleanliness, attitude of employees, and waiting time could be added to the primary attributes. This study focused on the general aspect of massage parlor services, which served as a benchmark for future studies. Hence, the investigated attributes were still considered adequate. Thus, researchers who wished to expand the study could opt for service quality models that itemized precise business characteristics.

## Conclusion

Academic studies about massage parlor business operations were inadequate despite the popularity of this business model, especially in Indonesia. Thus, this research analyzed consumer preference toward massage parlor services and features through conjoint design. A total of 212 respondents participated in an online questionnaire that was distributed using a convenience sampling approach. Six massage attributes were investigated, namely, gender of massage therapists, price of massage every session, duration of the massage, room type, type of massage, and Google review rating.

The results showed that the Google review rating was the most significant attribute (68.5%), followed by gender (12.4%), massage type (9.6%), room type (4.5%), duration (3.6%), and price (1.4%). At least a 4.6 Google review rating was the most favored one that should be maintained by massage parlors. In addition, massage parlors should employ more female massage therapists than male massage therapists. The reflexology massage type proved to be the most popular, followed by acupuncture with cupping, and the rest of the massage types were known to produce negative insights. Customers also preferred a common room, next was a couple room, and the least preferred room was the solo type. Consumers liked 120 minutes worth of massage as they felt more benefits through this duration. Then, the cheapest massage price (IDR 100,000 to IDR 150,000) was favored by most consumers. Furthermore, 27 stimuli were ranked based on their attributes’ importance values and levels’ utility estimates. Among 27 stimuli, the best combination was found as follows: female massage therapist, IDR 100k-150k price every session, 90 minutes massage duration, room type like a couple room with two beds, massage type of acupuncture and cupping, and massage parlors with greater than 4.6 Google review rating.

This study was deemed one of the first studies that explored holistic massage services in Indonesia. Unlike the previous studies that only focused on one massage service attribute or did not provide specific levels for evaluated attributes, the current research assessed six massage services coupled with 27 stimuli from massage service combinations. Smith et al. [13] only evaluated massage therapists’ gender and overlooked the importance of investigating other massage service attributes. In this study, six massage attributes were carefully assessed, including the gender of massage therapists. Corbos et al. [16] discussed pricing but did not categorize prices in numerical form. Thus, readers only had vague ideas about high price-high quality, high price-low quality, low price-low quality, and low price-high quality services. The present study specified Indonesia’s massage price ranges and consumers identified the price range that fit them the most. Moraska et al. [19] briefly mentioned duration and overlooked the importance of investigating duration’s effect on various massage types. Next, some studies were biased in selecting the room type as they pre-determined one room type instead of performing the experimental study in multiple room types [23–25]. Other studies focused on massage types’ effects on severe health cases [7, 26]. The saturation of severe cases in the massage industry was evident. Thus, the present study’s massage types covered various effects, such as health concerns, relaxation, pain relief, and boosting immunity. These catered to the overall well-being of consumers and were deemed not limited to certain diseases. Limited reviews were seen about Google review as most vaguely mentioned online consumer reviews without highlighting a specific platform [15, 17]. Therefore, the findings set a benchmark that could be utilized by other academicians to explore services and business operations offered by massage parlors worldwide.

The results could also be useful for massage parlor owners as they could adopt significant practical findings. Stakeholders were encouraged to focus on the most important attributes and levels, especially for massage parlors’ business owners who wanted to thrive in Indonesia. Moreover, the researchers presented in-depth managerial implications in the previous section that could gauge market segmentation and increase brand awareness. To highlight a few, massage parlors could maintain positive Google review ratings by starting with a well-trained massage therapist, clean and hygienic rooms, and even the smallest details such as oil selection. Marketing strategies entailed service customization based on consumer analysis and promotional discount reinforcement. Local government accreditation was also identified as another approach to establishing the credibility of massage parlors.
